# Ferroptosis and triple-negative breast cancer: Potential therapeutic targets

**DOI:** 10.3389/fonc.2022.1017041

**Published:** 2022-12-09

**Authors:** Na Xu, Baohong Li, Yong Liu, Cui Yang, Siqi Tang, William C. Cho, Zunnan Huang

**Affiliations:** ^1^ The First Dongguan Affiliated Hospital, Guangdong Medical University, Dongguan, Guangdong, China; ^2^ Key Laboratory of Big Data Mining and Precision Drug Design of Guangdong Medical University, Key Laboratory of Computer-Aided Drug Design of Dongguan City, Key Laboratory for Research and Development of Natural Drugs of Guangdong Province, School of Pharmacy, Guangdong Medical University, Dongguan, Guangdong, China; ^3^ Department of Clinical Oncology, Queen Elizabeth Hospital, Kowloon, Hong Kong SAR, China; ^4^ Marine Medical Research Institute of Guangdong Zhanjiang, Zhanjiang, Guangdong, China

**Keywords:** triple-negative breast cancer, ferroptosis, prognostic model, immune infiltration, drug prediction

## Abstract

**Purpose:**

Triple-negative breast cancer (TNBC) is an aggressive tumor with poor prognosis, it has higher recurrence and metastatic rates than other breast cancer subtypes. This study aims to investigate biomarkers and potential targets for TNBC related to ferroptosis through data mining and bioinformatics analysis. The findings may provide new insights for treating TNBC.

**Methods:**

The TNBC patients’ data from the Cancer Genome Atlas (TCGA) database were extracted for differential expression and prognosis analysis. Consensus genes obtained by intersecting differential expressed and ferroptosis-related genes was used to establish the prognostic model by the univariate and multivariate Cox analyses. Besides, TNBC data from the Gene Expression Omnibus (GEO) database was used to confirm the reliability of the prognosis model. Moreover, clinical information was analyzed by multifactorial independent analysis to identify independent prognostic factors. The expression of genes constituting the prognostic model was further validated using the Human Protein Atlas (HPA) database. Finally, the Comparative Toxicogenomic Data (CTD) database was used to explore possible treatment drugs for TNBC.

**Results:**

We obtained 13,245 differential expressed genes, and 177 consensus genes. 98 genes with prognostic implication were obtained by univariable Cox. Then, a prognostic model including 12 ferroptosis-related genes was constructed by multivariable Cox. The area under curve (AUC) value of the prognostic model for TNBC was 0.82. The GEO database validated that the model (AUC = 0.77) could predict the patient outcomes. The staining results of 10 out of 12 prognostic model genes in HPA database showed that their expression was consistent with our predictions. Clinical risk analysis indicated that risk score of patients could act as an independent prognostic factor. Finally, six drugs that may have interaction with 12 ferroptosis-related genes were obtained using the CTD database.

**Conclusion:**

The prognostic model composed of 12 ferroptosis-related genes could predict the prognosis of TNBC patients, and seven genes (ASNS, LAMP2, CAV1, DPP4, HELLS, TF, ZFP69B) could be potential new therapeutic targets for TNBC, and two drugs (1-methyl-3-isobutylxanthine, rosiglitazone) could act as potential therapeutic drugs for the treatment of TNBC.

## Background

Breast cancer is one of the most frequent malignancies and the common cause of cancer death in women (1). Breast cancer is a heterogeneous tumor with histological morphological and genomic differences. Based on genomic differences, it can be divided into molecular subtypes such as HER2, Lumina A, Lumina B, and triple-negative breast cancer (TNBC) (2). In TNBC, progesterone receptor (PR), estrogen receptor (ER) and human epidermal growth-factor receptor 2 (HER2) are all negatively expressed (3). Thus, conventional targeted therapies are ineffective in patients with TNBC. Compared with those of other breast cancer subtypes, TNBC patients have normally shorter survival time, higher risk of recurrence and invasion with the extension of treatment time and the development of drug resistance (4). In recent years, the platinum containing regimen has been the most studied and effective therapy for TNBC patients (4), and neoadjuvant chemotherapy has been considered as the preferred approach for the majority of the patients with early-stage TNBC due to the ability of increasing their prognosis and survival (5). However, Poggio et al. found that the platinum-based neoadjuvant chemotherapy was associated with a worse hematological toxicity (6). With the development of bioinformatics theory and emerging study of ferroptosis in cancer research area, clinical researchers gradually turned their focus to novel ferroptosis-related targets for TNBC treatment.

Ferroptosis is a new non-programmed apoptosis death process discovered by chemical screening (7). It is mainly caused by mitochondrial lipid peroxidation *via* iron-dependent accumulation of reactive oxygen species (ROS) (7). In recent years, induction of ferroptosis has appeared as a prospective therapeutic choice, especially for those malignancies that are resistant to conventional treatments (8). For examples, Ding et al. used breast cancer cells (MDA-MB-231, SUM159, BT-574) and found that DMOCPTL directly bound to the glutathione peroxidase 4 (GPX4) to up-regulate early growth response 1 (EGR1) in TNBC cells, leading to apoptosis of mitochondrial mediator and further inhibiting breast tumor growth (9). Song et al. used breast cancer cell lines (HS578t and MDA-MB-231) and found that the expression of GPX4 was increased in gefitinib-resistant cells, and when GPX4 was silenced, the expression of intracellular malignant malonaldehyde (MDA) and ROS increased and the expression of glutathione (GSH) decreased, which promoted the ferroptosis of cells, and then increased the sensitivity of cells to gefitinib (10). Wen et al. found that 18β-glycyrrhetinic acid (GA) promoted the production of ROS and reactive nitrogen species (RNS) by activating inducible nitric oxide synthase (iNOS), and then suppressed the activity of GSH and GPX4 by deactivating nicotinamide adenine dinucleotide phosphate oxidase (NADPH), thereby aggravating lipid peroxidation and triggering ferroptosis in breast cancer cells (MDA-MB-231) (11). Therefore, it is a new idea to regulate tumor progression by regulating ferroptosis.

Bioinformatics can be used to pre-screen differentially expressed genes and further construct a prognostic model, in which we can find the potential biomarkers related to the survival and prognosis of patients and discover the potential novel targets associated with tumors, providing additional support for research into disease mechanisms and new drugs. In this study, based on the public TNBC profiles from the Cancer Genome Atlas (TCGA), the Gene Expression Omnibus (GEO) database, and the clinical TNBC samples from the University of California Santa Cruz (UCSC) databases, we explored the potential oncogenesis and progression mechanisms of ferroptosis in TNBC from the perspectives of gene expression, prognostic model, biological pathways, immune infiltrations and target drugs. We believe this study offers novel characteristic biomarkers and medications for TNBC, broadens the clinical significance of the Cox model from prognosis prediction to precise therapy, and provides the researchers a new way of integrated bioinformatic analysis on predicting potential ferroptosis-related targets and drugs of TNBC.

## Materials and methods

### Data download

1,222 cases of breast cancer (including 113 normal cases and 1,109 tumor cases) were obtained from TCGA database (https://portal.gdc.cancer.gov/) and the clinical and phenotype data of breast cancer were downloaded from UCSC database (http://xena.ucsc.edu/). GSE31519 dataset was downloaded from GEO database (https://www.ncbi.nlm.nih.gov/geo/) and the ferroptosis-related genes were obtained from the FerrDB data (http://www.zhounan.org/ferrdb/current/) for subsequent analysis and verification.

### Data extraction

122 cases related to TNBC from 1109 cases of breast cancer were screened out by the clinical and phenotypic data from UCSC database. And 62 cases related to TNBC from GSE31519 dataset were screened out by the clinical information of GEO dataset.

### Acquisition of differential genes

TNBC data from the TCGA database were differentially analyzed and visualized using R version 4.05 (https://www.R-project.org, R Foundation for Statistical Computing, Vienna, Austria). Among them, the “limma” package was applied to obtain differentially expressed genes with adjusted *P*-value < 0.05, and |logFC| > 0, and the “ggrepel” and “ggplots2” packages were used to draw and visualize the volcano plot of the differential genes. Adjusted *P*-value took in account the false discovery rate (FDR), could help reduce the number of false positives.

### Univariate Cox regression analysis

The genes related to ferroptosis from the FerrDB database were intersected with the differential genes to obtain consensus genes, and the expression matrix of the consensus genes was extracted. Survival analysis was performed on the consensus gene using R. Using adjusted *P*-value (FDR) < 0.05 as the screening condition, the “survival” package was used to analyze OS survival data and obtain ferroptosis-related genes with prognostic value.

### The construction of prognostic model

Using the “survival” and “survminer” packages, the multivariate Cox analysis was performed on the ferroptosis-related genes with prognostic value. And then a prognostic model was established. Among the genes constituting the prognostic model, the genes with adjusted *P*-value (FDR) < 0.05 could exist as independent prognostic factors for TNBC. The risk scores of patients were obtained according to the expression and the correlation coefficients of the prognostic genes. And then the patients were separated into two risk groups (high and low) by the median of the risk scores, the survival rates of the two groups were also calculated. The ROC curve was drawn to verify the reliability of the prognostic model by “timeROC” package. The GEO data was utilized to verify the dependability of the prognostic model, and the “Pathology” and “Tissue” modules in the Human Protein Atlas (HPA) database (https://www.proteinatlas.org) were used to further confirm the protein expression of the genes.

### Analysis of clinical risk factor

For the purpose of further exploring the reliability of the prognosis model, the clinical data (Age, Stage, T, N, M and Risk Score of the model) from UCSC database were sorted, and the “survival” and “surviviner” packages were used for univariate and multivariate Cox analysis. Among them, the clinical factors with adjusted *P*-value (FDR) < 0.05 could exist as independent prognostic factors.

### Functional enrichment analysis

To explore the specific functions and mechanisms of ferroptosis-related genes in TNBC patients, the Gene Ontology (GO) and Kyoto Encyclopedia of Genes and Genomes (KEGG) analysis were acted on the expression matrices of the consensus genes by utilizing the “clusterProfiler”, “org.Hs.eg.db”, “enrichplot” and “ggplot2” packages. The GO functional annotations and KEGG pathways associated with the consensus genes were screened out with adjusted *P*-value (q-value) < 0.05, which was also calculated by taking the FDR into account to reduce the number of false positives.

### Immune infiltration analysis

To investigate the immune infiltration of cases, TNBC data from TCGA were immunized using the “ggplot2”, “reshape2”, “ggpubr” and “dplyr” packages with “ Cibersort.R “ data and 22 immune cell types (LM22) as background files (12). Among them, the Permutations coefficient was 1000, and the CIBERSORT output value RMSE was normalized to one to explore the different immune infiltration between the normal group and the tumor group (12).

### Potential drug prediction

To further study the role of genes, the “Discover” function of the Comparative Toxicogenomics Database (CTD) database (http://ctdbase.org/) was used to explore the compounds that interact with the genes constituting the prognostic model. And the compounds were arranged by “Interaction Count”. Compounds with Interaction count value greater than or equal to 45 were screened out as the examples of potential compounds related to TNBC treatment.

## Results

### Flowchart of the study

**Figure 1:**
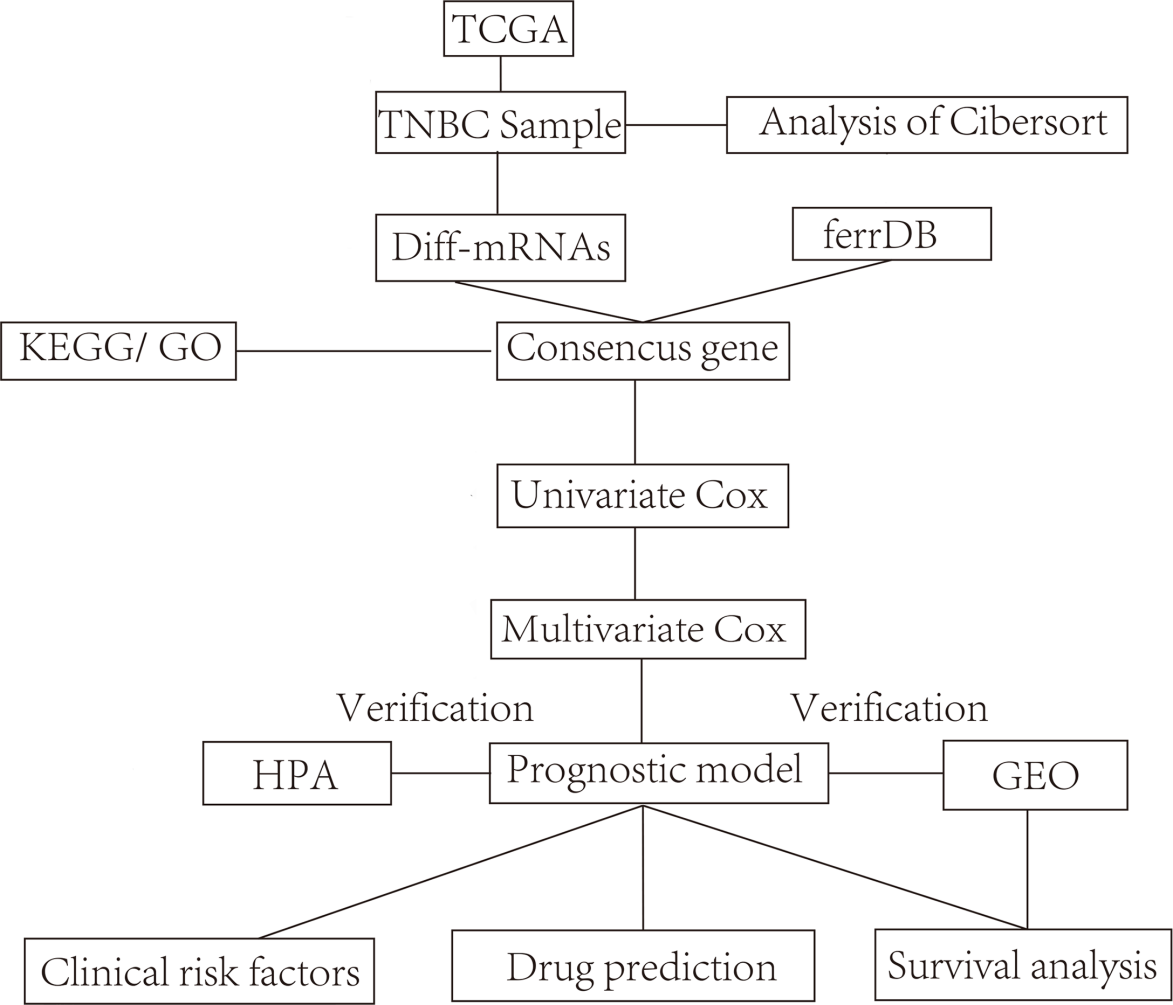
Study flowchart.

**Figure 1** shows the flowchart of the study in the identification of ferroptosis-related prognostic model related to the diagnosis and treatment of TNBC. In this study, the differentially expressed mRNAs (diff-mRNAs) were first screened out by the TNBC-related data from the TCGA database and the ferroptosis-related genes (ferrDB) were downloaded from the FerrDB database. Then, the consensus genes were obtained by the intersection of diff-mRNAs and ferrDB. Subsequently, the signal pathways and biological functions associated with the consensus genes were analyzed through the KEGG and GO databases respectively. Meantime, a prognostic model with the ability to predict the patients’ survival was constructed based on the consensus genes through univariate and multivariate Cox analyses and further validated using the dataset derived from GEO database. After that, the abnormal expressions of the prognostic model-related key genes were also verified in the HPA database and the clinic prognostic factors were analyzed using multifactorial independent analysis. Moreover, the immune infiltration of the immune cells in the normal and tumor samples related to TNBC was further evaluated and compared with each other *via* the CIBERSORT analysis tool. Finally, the potential drugs interacted with the model-related key genes were mined in the CTD database.

### Acquisition of differential genes

**Figure 2:**
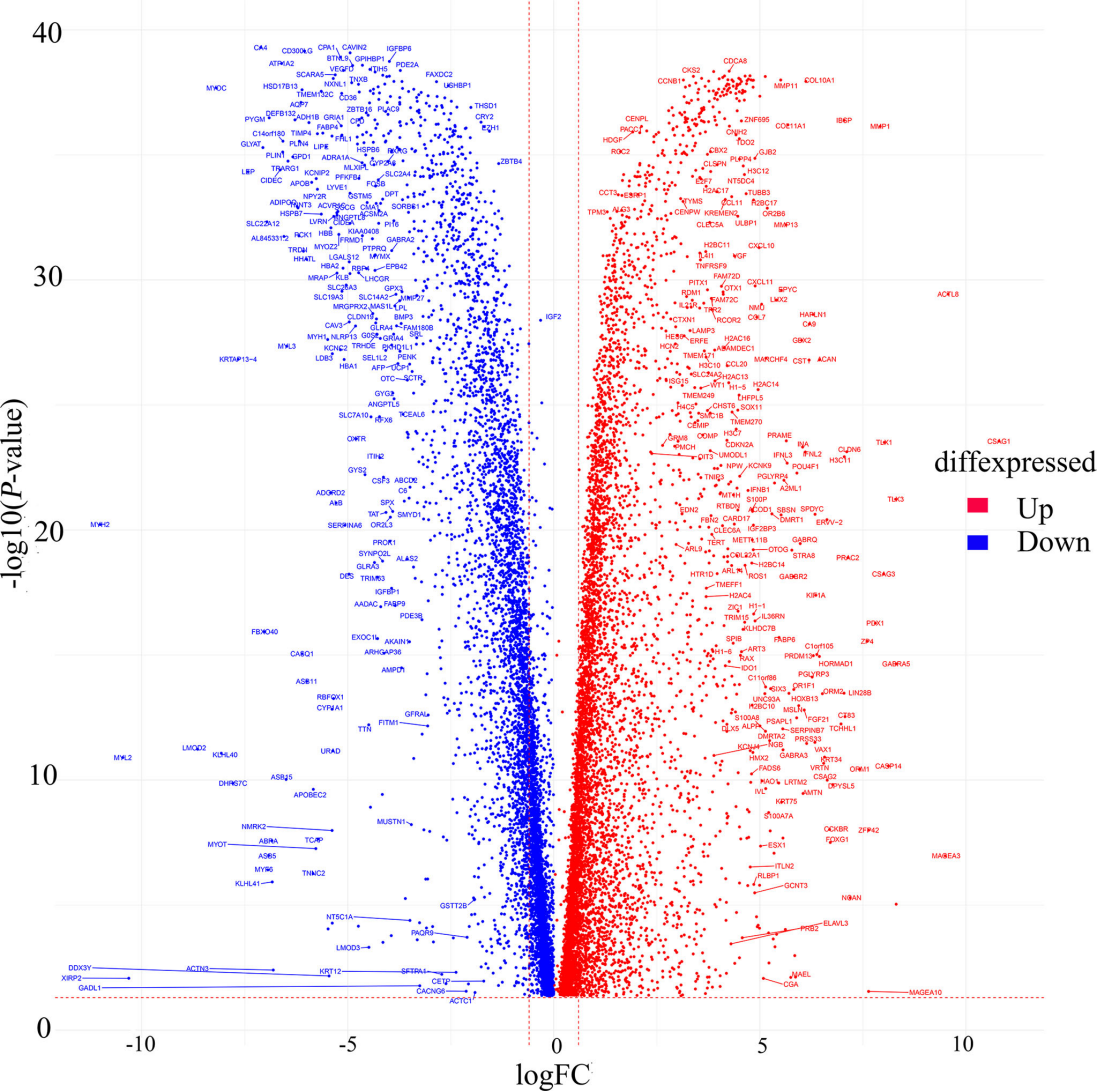
Volcano map of differentially expressed genes from TCGA. Red dots indicate up-regulated genes, blue dots indicate down-regulated genes.

235 relevant cases from the TCGA database (containing 113 normal cases, 122 TNBC cases) were performed for differential analysis. A total of 13,245 significant genes were obtained, of which 6,636 genes were down-regulated and 6,879 genes were up-regulated in expression (**Figure 2**).

### Univariate Cox regression analysis

A total of 259 ferroptosis-related genes were acquired from the FerrDB database, and 177 consensus genes were obtained by intersecting them with the differentially expressed genes obtained above. By univariate Cox analysis of consensus genes, 98 genes associated with the prognosis of TNBC patients were acquired. Subsequently, 29 prognostic genes were identified with adjusted *P*-value < 0.001 for subsequent multivariate Cox analysis (**Table 1**).

**Table 1:** 29 prognostic genes for subsequent multivariate Cox analysis.

Gene	HR	HR.95L	HR.95H	Adjusted *P*-value
STMN1	1.448843	1.222914	1.716512	1.82E-05
AURKA	1.347814	1.174372	1.546872	2.17E-05
RRM2	1.296064	1.147304	1.464112	3.06E-05
CDKN2A	1.264758	1.131071	1.414246	3.78E-05
CXCL2	0.781467	0.694867	0.878861	3.88E-05
TF	0.819579	0.740604	0.906977	0.000119
ZFP69B	1.567137	1.239917	1.980712	0.000170
DPP4	0.724702	0.612192	0.857889	0.000184
TFR2	1.290185	1.128821	1.474617	0.000186
PSAT1	1.371312	1.161152	1.619509	0.000199
PLIN4	0.884041	0.828449	0.943363	0.000200
KRAS	1.906233	1.353167	2.685347	0.000224
CA9	1.160919	1.070858	1.258555	0.000293
ANGPTL7	0.862074	0.794713	0.935143	0.000350
CHAC1	1.398498	1.162439	1.682493	0.000377
DUSP1	0.750088	0.639292	0.880085	0.000421
CAV1	0.782373	0.682309	0.897113	0.000440
ASNS	1.490974	1.191344	1.865962	0.000484
HELLS	1.385485	1.151039	1.667684	0.000567
LAMP2	1.968413	1.338305	2.895194	0.000581
MIOX	1.312865	1.123810	1.533724	0.000601
CDO1	0.846008	0.768909	0.930838	0.000604
IL33	0.819455	0.731169	0.918401	0.000618
SLC2A1	1.388151	1.150439	1.674981	0.000621
HMGB1	2.037045	1.354107	3.064418	0.000638
PRDX1	1.694457	1.249974	2.296996	0.000680
ENPP2	0.776785	0.670471	0.899956	0.000769
SLC7A11	1.312607	1.120006	1.538328	0.000780
ELAVL1	1.905369	1.301513	2.789392	0.000916

HR, hazard ratio.

### Functional enrichment analysis

**Figure 3:**
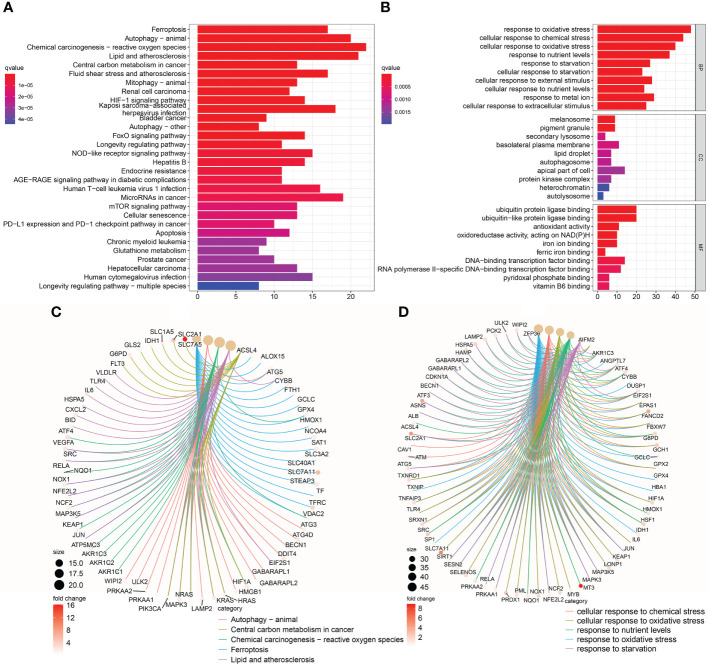
KEGG pathway and GO function analysis of consensus genes. **(A)** Histogram of KEGG pathway enrichment analysis. **(B)** Histogram of GO functional enrichment analysis. **(C)** Conceptual diagram of the gene network of the top five KEGG significantly enriched pathways. **(D)** Conceptual diagram of the gene network of the top five GO significantly enriched functions.

According to the results of KEGG pathway enrichment and GO functional analysis, 117 consensus gene of the TNBC sample mainly enriched in ferroptosis, autophagy-animal, chemical carcinogenesis-reactive oxygen species pathways (**Figure 3A**), response to oxidative stress, cellular response to chemical stress, cellular response to oxidative stress etc. in BP, and melanosome, pigment granule, secondary lysosome etc. in CC, ubiquitin protein ligase binding, ubiquitin-like protein ligase binding, antioxidant activity etc. in MF (**Figure 3B**). The top five most statistically significant (smallest adjusted *P*-value) pathways and functions were further demonstrated for gene enrichment, including ferroptosis, autophagy-animal (**Figure 3C**) and response to oxidative stress and cellular response to oxidative stress (**Figure 3D**), etc.

### Construction and validation of prognostic model

**Figure 4:**
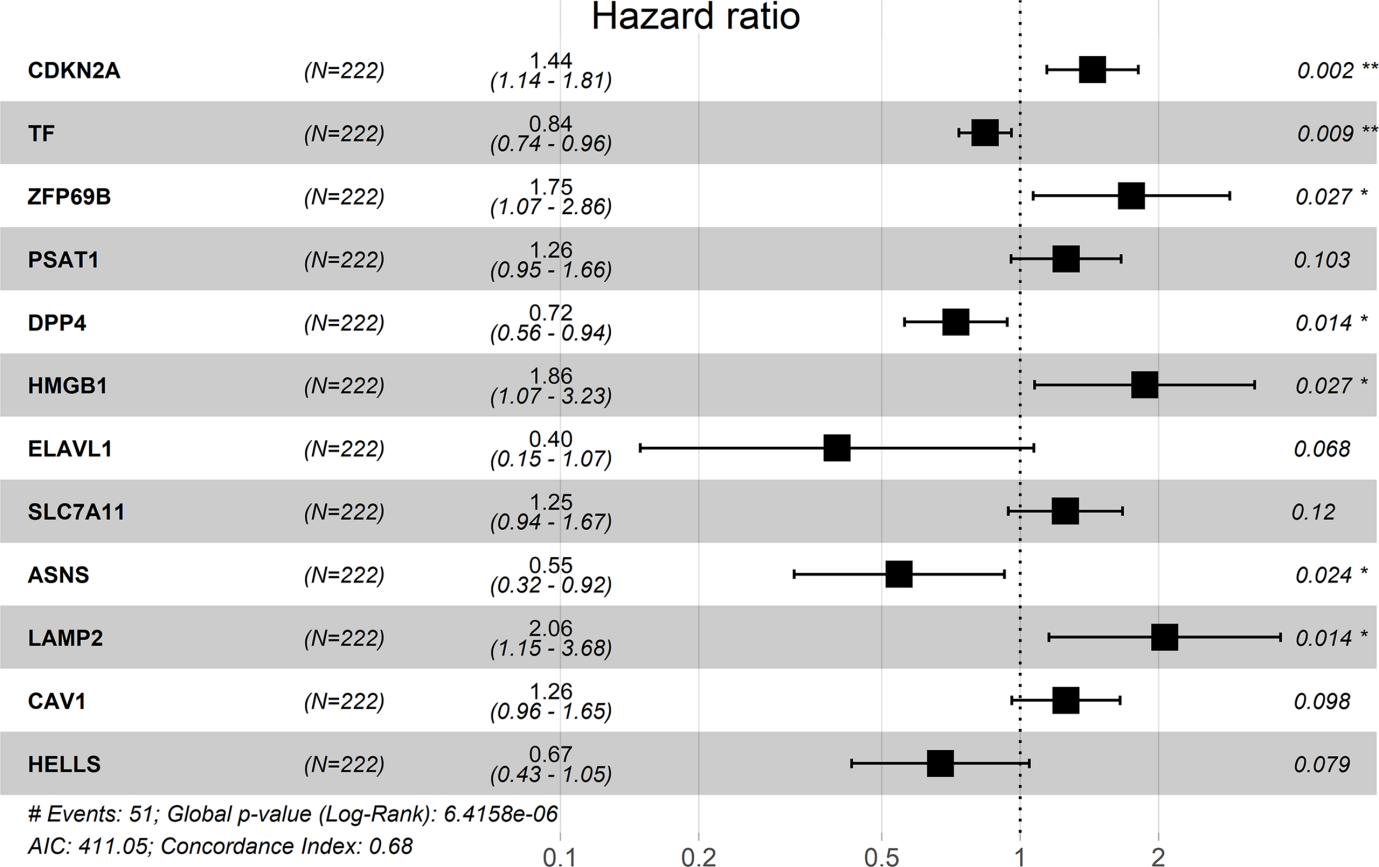
Risk forest plot of 12 prognostic genes. “*” represents adjusted *P*-value < 0.05, “**” represents adjusted *P*-value < 0.01.

**Figure 5:**
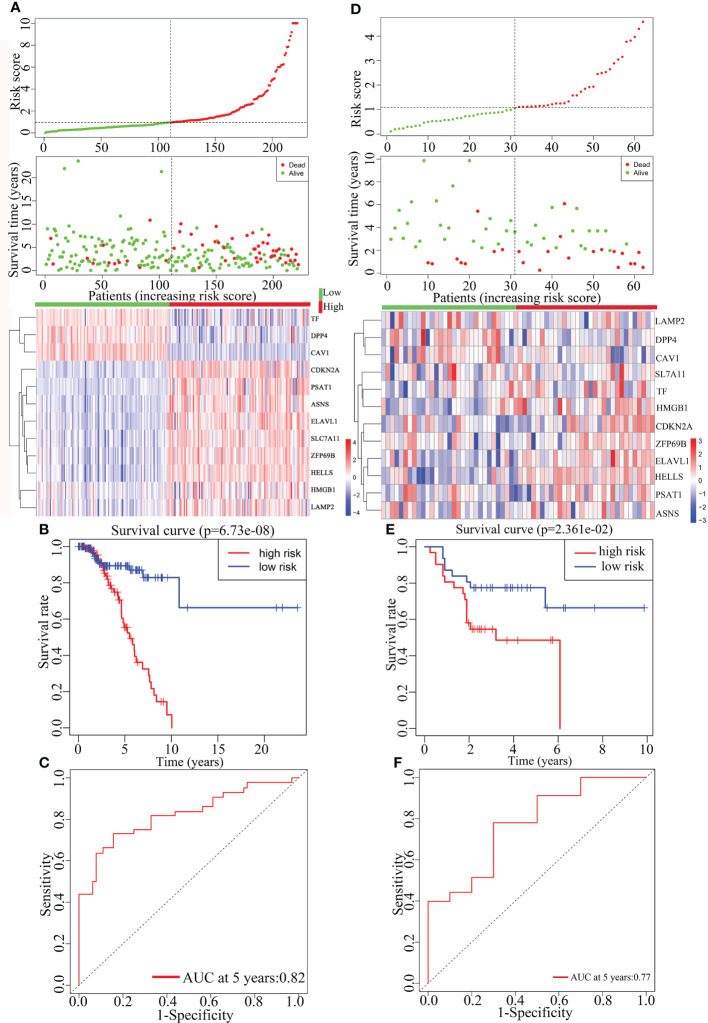
The construction and the validation of the prognostic model for TNBC patients. The data on the left were from TCGA data and the data on the right were from GEO data. From top to bottom were the risk score of patients, patient survival status, expression profiles of the 12 prognosis-related genes **(A, D)**, Kaplan-Meier curves for the high-risk and low-risk groups **(B, E)** and the ROC curves **(C, F)**.

Multivariate Cox analysis was performed on the above 29 genes to obtained 12 genes correlated with the prognosis of TNBC patients (**Table 2**), and the prognosis model was composed of them, including CDKN2A, TF, DPP4, LAMP2, ASNS, HMGB1, ZFP69B, ELAVL1, HELLS, CAV1, PSAT1 and SLC7A11 (**Figure 4**). Among them, CDKN2A, LAMP2, ASNS, HMGB1, ZFP69B, ELAVL1, HELLS, PSAT1 and SLC7A11 were up-regulated and TF, DPP4 and CAV1 were down-regulated in TNBC compared with normal tissues (**Table 2**). In addition, CDKN2A, TF, DPP4, LAMP2, ASNS, HMGB1, ZFP69B could serve as independent prognostic genes in this prognostic model (adjusted *P*-value < 0.05, **Figure 4**). Patients Risk score = (0.361879*CDKN2A) + (-0.17576*TF) + (-0.32256*DPP4) + (0.723853*LAMP2) + (-0.60588*ASNS) + (0.622896*HMGB1) + (0.556995*ZFP69B) + (-0.91682*ELAVL1) + (-0.39918*HELLS) + (0.228499*CAV1) + (0.229658*PSAT1) + (0.226121*SLC7A11). The risk status showed the number of patient deaths in the high-risk group was greater than that in the low-risk group (**Figure 5A**). In addition, the survival curves illustrated the survival rates of the low-risk group was remarkably higher than that of the high-risk group (3-year survival rate was 83.69% in high-risk group, 95% CI = 75.57% - 92.70%, and 89.30% in low-risk group, 95% CI = 82.90%- 96.20%; 5-year survival rate was 55.42% in high-risk group, 95% CI = 43.01% -71.40% and 87.00% in low-risk group, 95% CI = 79.40%- 95.20%) (adjusted *P* = 6.73e-08, **Figure 5B**). The ROC curve with an AUC value of 0.82 indicated that the prognostic model could forecast the prognosis of TNBC patients well (**Figure 5C**).

**Table 2:** Multivariate Cox analysis of 12 genes correlated with the prognosis of TNBC patients.

Gene	Coefficient	HR	z	LogFC	Adjusted *P*-value
CDKN2A	0.361879	1.436026	3.090199	4.201286	0.002000
TF	-0.175760	0.838816	-2.612710	-0.506460	0.008983
DPP4	-0.322560	0.724295	-2.464460	-1.168990	0.013722
LAMP2	0.723853	2.062365	2.445957	0.637841	0.014447
ASNS	-0.605880	0.545595	-2.255560	2.030095	0.024098
HMGB1	0.622896	1.864319	2.216083	0.463356	0.026686
ZFP69B	0.556995	1.745419	2.214205	1.573934	0.026815
ELAVL1	-0.916820	0.399787	-1.823890	1.019786	0.068169
HELLS	-0.399180	0.670871	-1.758600	2.265128	0.078646
CAV1	0.228499	1.256713	1.654111	-3.409020	0.098105
PSAT1	0.229658	1.258170	1.632409	2.819846	0.102593
SLC7A11	0.226121	1.253727	1.553083	2.491765	0.120403

HR, hazard ratio.

The prognostic model was further validated by the gene expression and the clinical data from the TNBC data in the GEO dataset GSE31519. In accordance with the TCGA data, there was an increase in the number of patient deaths as the risk score increased (**Figure 5D**). Besides, due to the less clear of the expression pattern between low- and high-risk samples in **Figure 5D**, their detailed characteristics, including the survival status, average survival time, tumor grade, lymph node meta, and the mean/median expression levels of 12 prognostic genes, were summarized in **Supplementary Table 1**, which clearly showed the consistent results derived from different patient samples stored in TCGA and GSE databases respectively on the whole. The survival curve displayed that the patient’s survival rate was higher in the low-risk group than that in the high-risk group (**Figure 5E**). And the ROC curve (AUC = 0.77) indicated that the prognostic model in the validation set also forecasted the prognosis of TNBC patients well, indicating the reliability and independence of the prognostic model (**Figure 5F**).

### Validation of the expression of gene constituting the prognostic model

**Figure 6:**
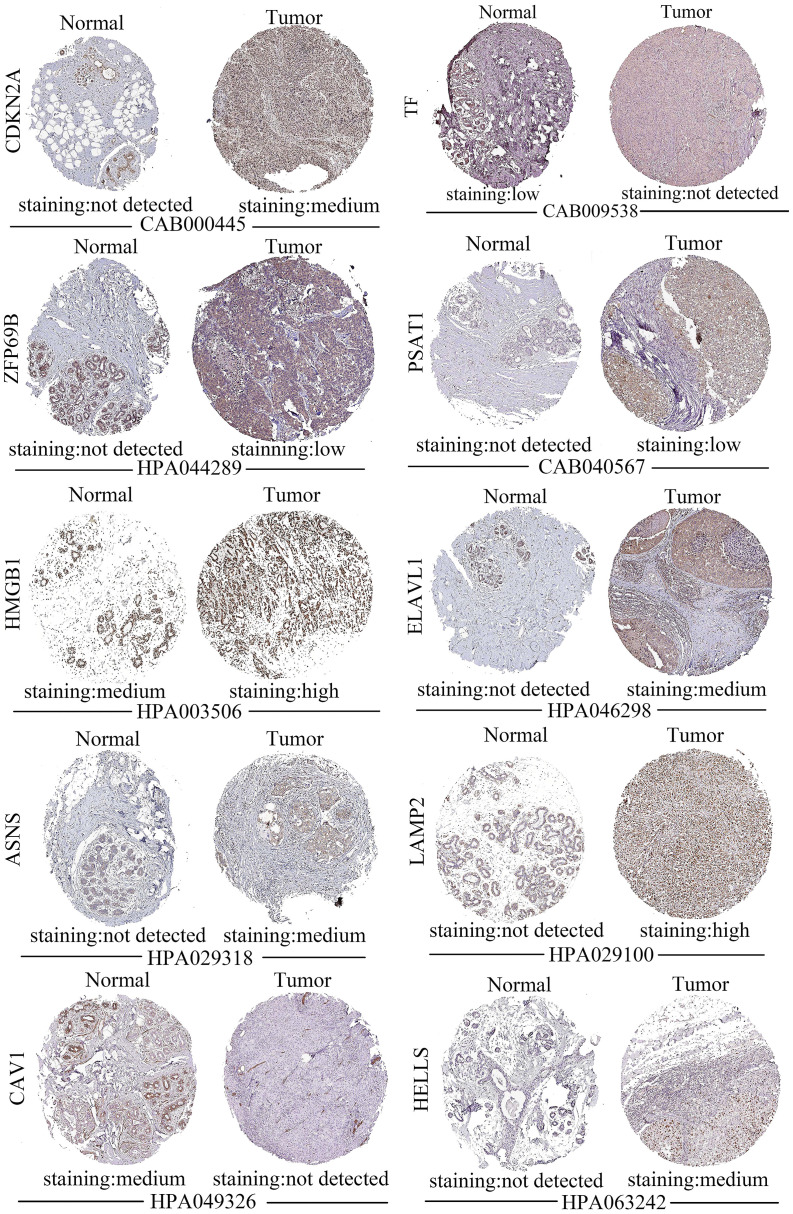
Staining of 10 out of 12 prognostic genes in ductal carcinoma tissue sections from the HPA database. In each set, normal tissue sections are shown on the left and tumor tissue sections are shown on the right.

**Figure 6** illustrated the staining of ductal carcinoma tissue for the genes constituting the prognostic model in the HPA database, and the left side denotes the normal group and the right side represents the tumor group. There were no associated proteins were detected in the tissue sections for DPP4 (date not shown) and no tissue sections for SLC7A11. The results of the HPA data showed that the tissue-stained sections for the remaining 10 prognostic model genes (CDKN2A, TF, ZFP69B, PSAT1, HMGB1, ELAVL1, ASNS, LAMP2, CAV1, HELLS) were consistent with our predictions (**Figure 6**). The HPA database only has sections of tissue classified according to pathological features, while TNBC is predominantly invasive ductal carcinoma (13), so we chose sections of ductal carcinoma tissue for this exploration. The staining of these tissue sections in the HPA database also demonstrated the accuracy of our computational predictions.

### Clinical risk factor analysis

Using the clinical data downloaded from the TCGA and UCSC databases, we conducted a multifactorial independent analysis to assess their clinical risk factors. The results (**Table 3**) showed that the adjusted *P*-value for the Risk Score in the multi-factor analysis was less than 0.05, indicating that the Risk Score value could exist as an independent prognostic factor. The adjusted *P*-value for the Risk Score in the univariate analysis was greater than 0.05, which might be due to the multicollinearity caused by the influence of confounding factors (HR value including 1). In addition, the clinical risk factor N (Node, referring to the lymph node metastasis of the tumor) had an adjusted *P*-value of less than 0.05 in both multifactorial as well as univariate analyses, and its HR was greater than 1, suggesting that it may play a notable role in the tumor progression of TNBC patients.

**Table 3:** Univariate and multivariate analysis of clinical risk.

	Univariate				Multivariate			
Clinical factor	HR	HR.95L	HR.95H	Adjusted *P*-value	HR	HR.95L	HR.95H	Adjusted *P*-value
Age	0.98649	0.94951	1.02491	0.48535	0.98538	0.94824	1.02397	0.45226
Stage	1.60457	1.02570	2.51014	0.03835	1.28376	0.28072	5.87081	0.74742
T	1.27942	0.78431	2.08707	0.32369	1.03782	0.27569	3.90683	0.95623
M	0.83587	0.34225	2.04144	0.69394	0.59525	0.22579	1.56923	0.29422
N	2.88021	1.68402	4.92606	0.00011	3.63453	1.47796	8.93790	0.00494
Risk Score	1.22551	0.98369	1.52679	0.06979	1.35962	1.06008	1.74379	0.01554

T, tumor; M, metastasis; N, node.

### Immune infiltration analysis

**Figure 7:**
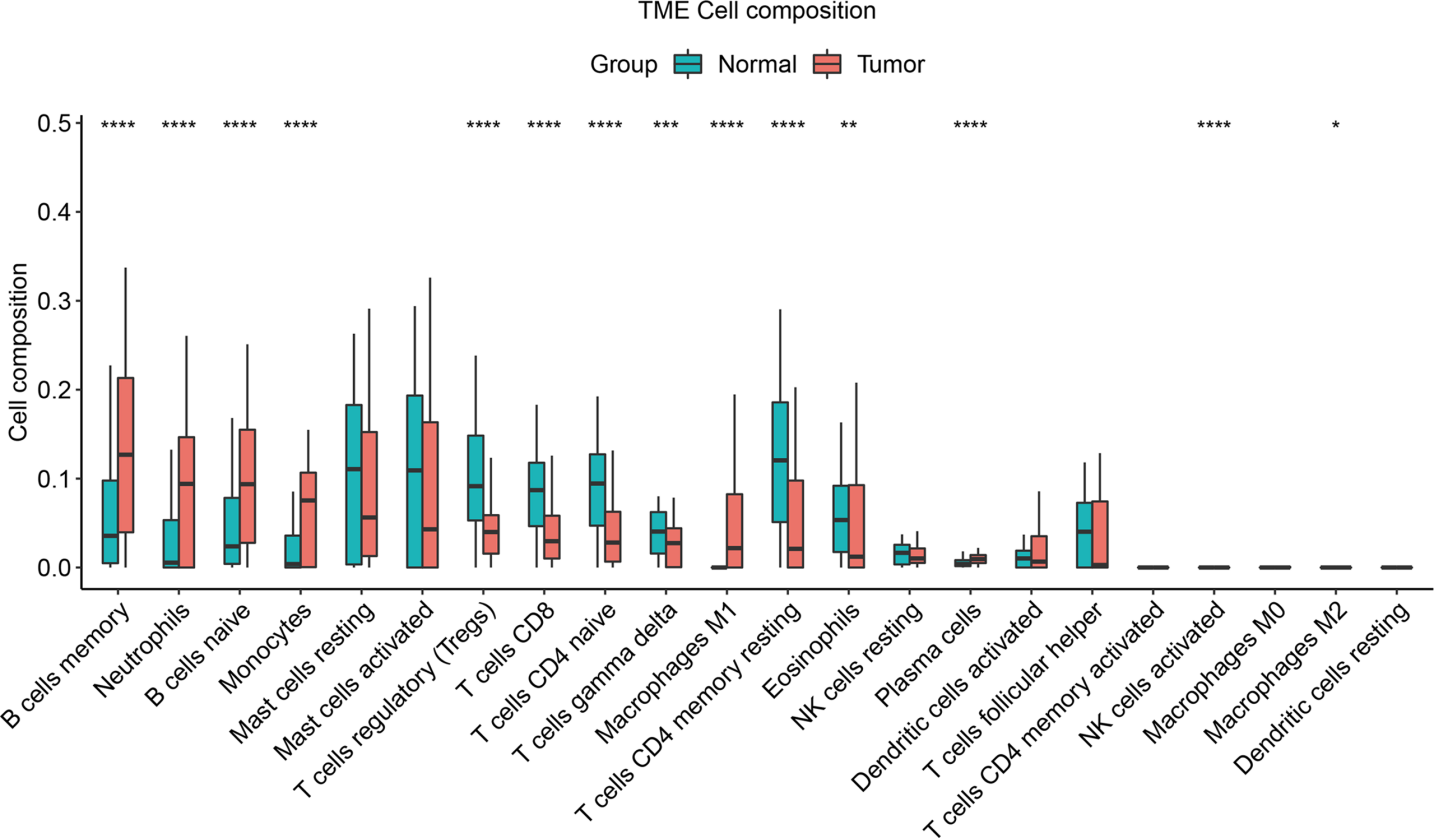
Immune infiltration composition of the 22 immune cells in the cases was predicted by the CIBERSORT algorithm for the 113 normal cases, 122 TNBC cases. Green denotes the normal group and the red denotes the tumor group. “*” represents adjusted *P*-value < 0.05, “**” represents adjusted *P*-value < 0.01, “***” represents adjusted *P*-value < 0.005, “****” represents adjusted *P*-value < 0.001.

Based on the immune infiltration of 22 immune cells in the 113 normal cases, 122 TNBC cases, we could see that the tumor group of the TNBC data had significantly lower proportions of T cells regulatory (Tregs), T cells CD8, T cells CD4 naïve, T cells gamma delta, T cells CD4 memory resting, and eosinophils (*P*-value < 0.01), while the proportions of B cells memory, neutrophils, B cells naïve, monocytes, macrophages M1, and plasma cells were notably increased (*P*- value < 0.001) (**Figure 7**).

### Potential drug prediction

**Figure 8:**
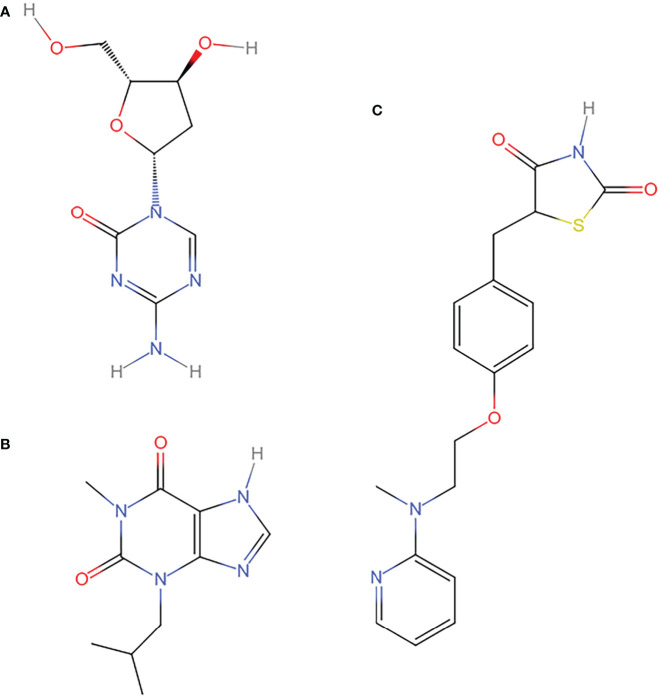
Compounds with potential therapeutic effect on key genes. **(A)** decitabine **(B)** 1-methyl-3-isobutylxanthine **(C)** rosiglitazone. The structures of the compounds with potential therapeutic value from the CTD database were redrawn by Indraw after taking the reference of those shown in the PubChem database.

2442 compounds with interaction value ranged from 1 to 50 were obtained from the prediction of CTD (**Supplementary Table 2**). **Table 4** listed the top six compounds interacting with the key genes: decitabine (**Figure 8A**), dexamethasone, lipopolysaccharides, iron, 1-methyl-3-isobutylxanthine (**Figure 8B**), rosiglitazone (**Figure 8C**). Among them, decitabine, dexamethasone had an interaction value of 50, lipopolysaccharides, iron had an interaction value of 46 and 1-methyl-3-isobutylxanthine, rosiglitazone had an interaction value of 45, these compounds may have a potential effect on TNBC.

**Table 4:** Compounds interacting with key genes from the CTD database.

Chemical Name	Chemical ID	Interaction Count	Structure
Decitabine	D000077209	50	**Figure 8A**
Dexamethasone	D003907	50	–
Lipopolysaccharides	D008070	46	–
Iron	D007501	46	–
1-Methyl-3-isobutylxanthine	D015056	45	**Figure 8B**
Rosiglitazone	D000077154	45	**Figure 8C**

## Discussion

TNBC is the most aggressive molecular subtype of breast cancer and it is highly heterogeneous in terms of the tumor microenvironment. Despite advance in the treatment, the prognosis of TNBC patients remains poor (14). Therefore, it is very important to explore potential therapeutic or therapeutic-promoting biomarkers and drugs. This study is one of the pioneer studies to investigate the prognosis of TNBC from the perspective of ferroptosis. In this study, we used TCGA and UCSC databases and their molecular characteristics to screen out relevant cases (113 normal cases and 122 TNBC cases), and performed differential analysis (13,245 differentially expressed genes). Subsequently, 177 consensus gene were obtained by intersecting the differentially expressed genes with the ferroptosis-related genes. Using consensus genes and univariate Cox, 98 consensus gene with prognostic value were screened out. Finally, using multivariate Cox and consensus genes with adjusted *P*-value < 0.01, we successfully constructed a prognostic model composed of ferroptosis-related genes (the AUC was 0.82). We used the relevant dataset GSE31519 of GEO database to confirm the prognostic model (the AUC was 0.77), and also used the HPA database to validate the expression of the genes constituting the prognostic model. Since TNBC and ductal carcinoma are not identical, ductal carcinoma tissue sections cannot fully represent TNBC data, which might be the reason for the absence of DPP4 protein staining in the sections. And the results indicated that the expressions of the genes were basically consistent with our prediction by calculation.

We checked the relevant literature to explore the expression and the possible mechanisms in ferroptosis related genes (**Tables 5**, **6**). As shown in **Table 5**, experimental studies have shown that CDKN2A, PSAT1, HMGB1, ELAVL1, and SLC7A11 were up-regulated in TNBC, ASNS and LAMP2 were up-regulated in breast cancer and CAV1 was down-regulated in breast cancer, and HELLS was up-regulated in other tumors. In addition, computational prediction indicated that DPP4 was down-regulated in breast cancer, TF was down-regulated in other tumors and ZFP69B was up-regulated in other tumors. The expression trends of all these genes were aligning with our models. Thus, all these studies demonstrated the reliability of our computational predictions.

**Table 5:** The abnormal expressions of key genes constituting the prognostic model.

Gene	Feature	Remark	Reference
CDKN2A	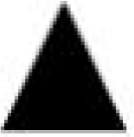	CDKN2A is also known as P16. Yoon et al. illustrated that the expression of p16 was up-regulated in TNBC sample by using tissue microarray, and a significant co-expression correlation existed between p16 and SRY-box transcription factor 10 (SOX10) but not in non-TNBC cases.	(15)
PSAT1	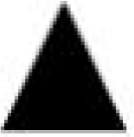	Metcalf et al. showed that the expression of PSAT1 was up-regulated in TNBC by using cell and qPCR, and the expression of PSAT1 increased with the increase of the clinical grade of TNBC.	(16)
HMGB1	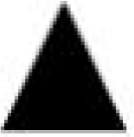	Lee et al. found that HMGB1 was highly expression in TNBC by using patient tissues and immunohistochemistry, and the expression of HMGB1 was notably correlated with tissue grade, abundant tumor infiltrating lymphocytes and CD8 positive cells.	(17)
ELAVL1	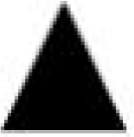	ELAVL1 is also known as HuR. Mehta et al. verified that HuR was up-regulated in TNBC cells compared with normal cells using cellular and western-blot analysis, and knockdown of HuR induced oxidative stress and DNA damage in cells, and further enhanced sensitivity of TNBC cells to radiotherapy.	(18)
SLC7A11	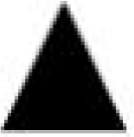	Bai et al. illustrated that the expression of SLC7A11 was up-regulated in TNBC cells compared with normal cells by immunohistochemistry test and the overexpression of SLC7A11 was connected with the promotion of glutathione biosynthesis, which further contributed to a radioresistance in TNBC.	(19)
ASNS	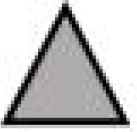	Qin et al. showed that ASNS highly expressed in breast cancer by using cell and qPCR, and the high expression of ASNS was correlated with poor prognosis.	(20)
LAMP2	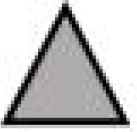	Damaghi et al. illustrated that LAMP2 highly expressed in breast cancer by using proteomics and qRT-PCR, which was positively associated with the course of disease.	(21)
CAV1	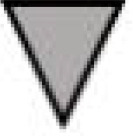	Witkiewicz et al. found that down-regulated CAV1 in breast cancer was correlated to poorer overall survival by using patient tissues and immunostaining.	(22)
HELLS	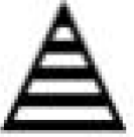	Hou et al. showed that HELLS was up-regulated in pancreatic tissues and correlated with advanced clinical stage and poor prognostic by using qRT-PCR. Knockdown of HELLS could lead to tumor cell arrest and increase sensitivity to cisplatin.	(23)
DPP4	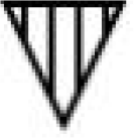	Choy et al. illustrated that DPP4 was notably down-regulated in breast cancer through comparative study using multiple public databases, and the down-expression of DPP4 may be related to the poor prognosis of patients.	(24)
TF	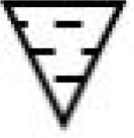	Hong et al. found that TF was down-regulated in melanoma by comparing the GSEA databases, and their study showed that the lipidogen regulator Sterol-regulatory element binding proteins (SREBP2) could directly induce the transcription of the TF, thereby decreasing the intracellular iron pool, the activity of oxygen and lipid peroxidation, and inhibiting ferroptosis in cells.	(25)
ZFP69B	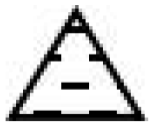	Zheng et al. showed that the expression of ZFP69B was up-regulated in gastric cancer by data mining through TCGA and GEO database, and a prognostic model composed of ZFP69B they further built could well forecast the prognosis of patients.	(26)

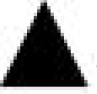
Gene was confirmed to be up-regulated by experiment in TNBC;

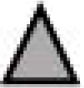
Gene was confirmed to be up-regulated by experiment in breast cancer;

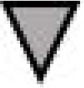
Gene was confirmed to be down-regulated by experiment in breast cancer;

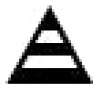
Gene was confirmed to be up-regulated by experiment in other cancers;

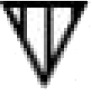
Gene was confirmed to be down-regulated by calculation in breast cancer;

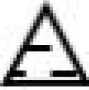
Gene was confirmed to be up-regulated by calculation in other cancers;

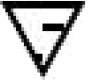
Gene was confirmed to be down-regulated by calculation in other cancers。

**Table 6:** The possible ferroptosis mechanism of 12 key genes.

Gene	Remark	Mechanism of ferroptosis	Reference
CDKN2A	CDKN2A is also known as P14ARF. Zhang et al. indicated that up-regulated P14ARF could promote ferroptosis in breast cancer cells by restraining nuclear factor erythroid 2-related factor 2 (NRF2)-mediated transcription of SLC7A11.	antioxidant defence	(27)
PSAT1	Jin et al. showed that under the condition of glutaminase deficiency, highly expressed activating transcription factor 4 (ATF4) upregulated the expression of PSAT1, which then led to the proliferation of breast cancer cells.	antioxidant defence	(28)
	Harding et al. indicated that ATF4 was an important regulator of the SLC7A11 subunit in the cystine/glutamate antiporter system (systerm XC-), and the deletion of ATF4 could lead to oxidative iron-dependent cell death.		(29)
SLC7A11	Lee et al. illustrated that up-regulated SLC7A11 could introduce cystine into cells, then promote GSH biosynthesis and GPX4 expression, thereby reducing the sensitivity of breast cancer cells to ferroptosis inducers.	antioxidant defence	(30)
ASNS	Ye et al. indicated that up-regulated ASNS activated ATF4, and then inhibited the cell death by oxidative stress in colorectal tumor cells.	antioxidant defence	(31)
LAMP2	LAMP2 is available in three isotypes: LAMP2A, LAMP2B and LAMP2C. Saha et al. showed that up-regulated LAMP2A reduced the oxidative modification of cellular proteins in breast cancer, and the inhibition of LAMP2A stimulated the accumulation of chaperone autophagy substrate GAPDH, AKT serine/threonine kinase 1 (AKT1) phosphorylation, ROS production and the induction apoptosis in breast cancer cells.	antioxidant defence	(32)
CAV1	Sotgia et al. indicated that matrix down-regulated CAV1 induced metabolic reprogramming of breast cancer cells, making them switch from oxidative mitochondrial phenotype to glycolytic phenotype. The down-regulated CAV1 created a hypoxic environment by increasing NO and ROS, stimulating the activation of HIF- 1α and NF-κB, and induced mitochondrial autophagy and oxidative mitochondrial metabolism in adjacent cells, thereby promoting cancer growth and metastasis.	antioxidant defence	(33)
ZFP69B	ZFP69B is also known as ZNF643. Dixon et al. indicated that the ferroptosis inducer Erastin induced the mRNA expression of ZNF643 by inhibiting system XC-, caused the depletion of cystine in lung cancer cells, and finally leading to the ferroptosis of cells.	antioxidant defence	(34)
ELAVL1	Lin et al. showed that highly expressed ELAVL1 could directly bind to the mRNA 3’-UTR of SLC7A11 to improve its stability and expression, and then increase the resistance of gastric cancer cells to ferroptosis.	antioxidant defence	(35)
TF	Hong et al. indicated that decreased of TF expression increased the pool of labile free iron, enhanced ROS and lipid peroxidation, and then reduced the sensitivity of melanoma cells to ferroptosis.	Iron toxicity	(25)
HELLS	HELLS is also known as LSH. Jiang et al. showed that high levels of LSH promoted the expression of lipid metabolism-related gene (GLUT1, SCD1 and FADS2), resulted in the reduction of intracellular lipid ROS, and then inhibited ferroptosis in lung cancer cells.	lipid peroxidation	(36)
	Jiang et al. showed that highly expressed LSH could inhibit Erastin-induced ferroptosis by reducing the intracellular concentration of iron in lung cancer cells.	Iron toxicity	(36)
HMGB1	Liu et al. indicated that overexpressed HMGB1 could induce autophagy in breast cancer cells.	lipid peroxidation	(37)
	Wen et al. showed that autophagy could promote the release and the acetylation of HMGB1 in ferroptosis.		(38)
	Fan et al. indicated that overexpressed HMGB1 could inhibit the ferroptosis of cell through TLR4-induced activation of the intracellular reductant NAD(P)H, which was necessary for the elimination of lipid peroxidase.		(39)
DPP4	Xie et al. showed that in the absence of TP53, DPP4 combined with NOX1 (NAPDH oxidase 1) to trigger membrane-associated DPP4-mediated lipid peroxidation, eventually leading to ferroptosis in colorectal cancer cells.	lipid peroxidation	(40)

It could be seen from **Table 6** that CDKN2A, PSAT1, SLC7A11, ASNS, LAMP2, CAV1, ZFP69B and ELAVL1 may contribute to the process of ferroptosis in cells through the “antioxidant defence”; TF may contribute to the process of ferroptosis in cells through the “iron toxicity”; HMGB1 and DPP4 may participate in the process of ferroptosis in cells through the “lipid peroxidation”; HELLS may contribute to the process of ferroptosis in cell through “iron toxicity” and “lipid peroxidation”. The mechanism of these genes in ferroptosis could further help the treatment of TNBC from the perspective of ferroptosis.

From the above, it could be seen that the abnormal expression of ASNS, LAMP2, CAV1, DPP4 in triple-negative breast cancer and HELLS, TF, ZFP69B in breast cancer have not been experimentally verified, and these genes could exist as potential treatment biomarkers of TNBC. In addition, the possible ferroptosis mechanisms of CDKN2A, PSAT1, SLC7A11, LAMP2, CAV1, and HMGB1 in TNBC and ASNS, ZFP69B, ELAVL1, TF, HELLS, and DPP4 in breast cancer have not been reported. The expression of these genes in TNBC and the possible mechanism of ferroptosis deserve further exploration, which will supply new scientific guidance for the treatment of TNBC patients.

In order to further explore the role of drugs interacting with genes in TNBC, we also queried the literature related to drugs (**Table 7**). As shown in **Table 7**, decitabine, rosiglitazone and 1-methyl-3-isobutylxanthine (**Figure 8**) could inhibit the development of tumor cells. In the process of inducing ferroptosis in tumor cells, decitabine may play the role of “antioxidant defence” and “lipid peroxidation”; rosiglitazone may play the role of “lipid peroxidation”; 1-methyl-3 -Isobutylxanthine may play a role through “antioxidant defence”. These studies may provide help for drug-induced ferroptosis in TNBC cells.

**Table 7:** Literature queries on drugs interacting with key genes.

Drug	Remark	Mechanism of ferroptosis	Reference
Decitabine	Yu et al. indicated that decitabine increased the sensitivity of TNBC cells to DNA methyltransferase (DNMT) by inducing ubiquitination and degradation of the E3 ligase TNF receptor-associated factor 6 (TRAF6).	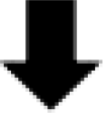	(41)
	Nakajima et al. illustrated that decitabine induced the expression of the B-cell lymphoma-2 (Bcl2) family protein NOXA.	antioxidant defence	(42)
	Ohshima-Hosoyama et al. indicated that NOXA could promote oxidative stress-induced cell death in medulloblastoma cell lines.		(43)
	Brglez et al. found that decitabine induced mRNA expression of phospholipase A2-related proteins Phospholipase A2 group IIA (PLA2G2A), Phospholipase A2 group III (PLA2G3) and Phospholipase A2 group X (PLA2G10) in TNBC, which in turn led to cell apoptosis.	lipid peroxidation	(44)
Rosiglitazone	Mody et al. indicated that rosiglitazone inhibited the viability and DNA synthesis of breast cancer cells, which in turn enhanced the sensitivity of tumor cells to tumor necrosis factor alpha (TNFα) and ultimately induced cell apoptosis.	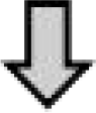	(45)
	Ishay-Ronen et al. showed that rosiglitazone in combination with the MEK inhibitor trametinib enhanced epithelial differentiation and adipogenesis *in vivo* and *in vitro*, thus promoting the conversion of invasive breast cancer cells into adipocytes, and ultimately inhibiting the metastasis of cancer cells.	lipid peroxidation	(46)
1-Methyl-3-isobutylxanthine	1-Methyl-3-isobutylxanthine is also known as 3-isobutyl-1-methylxanthine (IBMX). Lv et al. indicated that IBMX decreased the proliferation and invasion of glioma stem cells by activating the cAMP signaling pathway and inhibiting the mitogen-activated protein kinases (MAPK) signaling pathway.	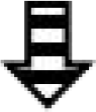	(47)
	Crumpton et al. found that IBMX induced the production of reactive oxygen in breast cancer cells and enhanced the sensitivity of cells to other oxidants, which in turn led to apoptosis.	antioxidant defence	(48)
	Lee et al. indicated that IBMX promoted tyrosinase activity and activated melanin production.	antioxidant defence	(49)
	Hill et al. indicated that melanin could generate destructive reactive oxygen and enhance DNA damage, leading to cell death.		(50)

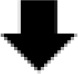
represents that the drug could inhibit the progression of TNBC by experiment.

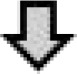
represents that the drug could inhibit the progression of the breast tumor by experiment.

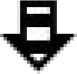
represents that the drug could inhibit the progression of other tumor by experiment.

As for dexamethasone, iron and lipopolysaccharide, studies have illustrated that the three can promote the development of tumors. For example, studies have shown that dexamethasone induced the expression of Krüppel-like factor 5 (KLF5) in a time- and dose-dependent manner, thereby reducing the sensitivity of TNBC to the chemotherapeutic drugs paclitaxel and cisplatin (51); excess iron could upregulate the expression of the inflammatory cytokine interleukin-6 (IL-6), which in turn triggered Janus kinase 2 (JAK2)/signal transducer and activator of transcription 3 (STAT3) signaling, ultimately leading to epithelial-mesenchymal transition (EMT) and migration of TNBC cells (52); lipopolysaccharide could up-regulate the expression of protein kinase (TOPK) in breast cancer cells, and then induce the migration and invasion of breast cancer (53).

At the same time, we are also interested in the pathways and biological function enriched in prognostic model genes, so as to lay a foundation for further exploration of the mechanism of TNBC-related ferroptosis. Han et al. showed that overexpression of ATF4 could increase protein synthesis, leading to oxidative stress and cell death (54), and that activation of ATF4 could trigger the transcription of SLC7A11 (19). Nah et al. indicated that phosphorylated CAV1 could trigger autophagy by binding to the Beclin 1 (BECN1)/VPS34 complex under oxidative stress (55). And the BECN1 could be phosphorylated under the stimulation of AMPK, then the phosphorylated BECN1 could promote ferroptosis by regulating the cysteine and glutamate anti-transporter system (56). These studies suggested that autophagy, oxidative stress and post-translational modifications played important roles in ferroptosis, which were consistent with the KEGG pathway and GO functions of “autophagy”, “oxidative stress” and “ubiquitin ligase binding” enriched by consensus genes in the sample data. Therefore, further exploration of pathways and functions could better explore the role of ferroptosis in TNBC, and provide new ideas and guidance for the treatment of TNBC.

The tumor immune microenvironment played a notable role in the mechanisms of drug resistance, and the response of TNBC to drugs was mainly controlled through the acquisition of signals from the tumor immune microenvironment (57). Studies have shown that substances in the tumor microenvironment interacted with the genes. For example. Wang et al. showed that interferon gamma (IFNγ) discharged from CD8 T cells reduced the expression of SLC7A11 and SLC3A2, inhibited the uptake of cystine by tumor cells, which in turn enhanced ferroptosis -specific lipid peroxidation in cancer cells, and increased ferroptosis contributed to the anti-tumor effect of immunotherapy (58). Xu et al. indicated that GPX4 could inhibit lipid peroxidation and ferroptosis in Treg cells, which in turn maintained the activation of Treg cell (59). In addition, some studies have shown that drugs could also alter the components of the tumor immune microenvironment. Bunt et al. illustrated that rosiglitazone in combination with gemcitabine could regulate T-cell populations by increasing circulating CD8+ T cells and intra-tumor CD4+ cells and CD8+ T cells, thereby reducing the progression and metastasis of pancreatic tumor, and further enhancing cell apoptosis (60). Grader-Beck et al. indicated that 1-methyl-3-isobutylxanthine increased endogenous cAMP, thereby inhibiting CD3 and CD28 mediated T cell activation and cytokine production (61). These studies demonstrated that ferroptosis could be promoted by altering the tumor microenvironment. Therefore, understanding the ferroptosis caused by changes in TNBC tumor immune infiltration through various pathways could further help us to explore the treatment of TNBC from the perspective of ferroptosis.

There are a few limitations in this study. The TNBC data were from the publicly available TCGA, GEO and UCSC databases, which means that the samples included in this study were not strictly randomized, and this may increase the sample error in statistics. Besides, only the top six out of 2442 compounds targeting to the genes of Cox model were analyzed as examples, the rest of them remains to be further investigated. In addition, the partial outcomes obtained by prognostic genes, models, signaling pathways and drugs related to ferroptosis, which provided the potential predictability of this study, need further experimental research and clinical verification. However, our study systematically explored the ferroptosis-related prognostic genes, their correlated drugs, immune infiltration and biological functional mechanisms in TNBC, which may provide new insights into ferroptosis-based targeted therapy for this cancer.

## Conclusion

In this study, we successfully constructed a prognostic model, which could well predict the prognosis of patients (AUC = 0.82). Seven genes (ASNS, LAMP2, CAV1, DPP4, HELLS, TF, ZFP69B) could be present as potential therapeutic biomarkers for TNBC. Two drugs (rosiglitazone and 1-methyl-3-isobutyl-cryptoxanthine) could be used as potential therapeutic agents for TNBC. The possible ferroptosis mechanisms of seven genes and two drugs could contribute to the induction of ferroptosis in TNBC cells, and supply help for the treatment of TNBC.

## Data availability statement

The original contributions presented in the study are included in the article/**Supplementary Materials**. Further inquiries can be directed to the corresponding authors.

## Author contributions

ZH, WC, NX, and BL contributed to the design and conception of the study. NX and YL did information retrieval and analysis. NX, BL and ST wrote the manuscript. NX and CY created tables and figures. ZH, WC and BL guided manuscript writing and revised the manuscript. ZH provided financial support. All authors contributed to the article and approved the submitted version.
