# Supplementary material for: Ferroptosis and triple-negative breast cancer: Potential therapeutic targets
Source: Front Oncol. 2022 Dec 9;12:1017041. doi: 10.3389/fonc.2022.1017041 (PMC9780505; doi:10.3389/fonc.2022.1017041)
Supplement: Supplementary file 1 [file Table_1.docx]

**Ferroptosis and triple-negative breast cancer: potential therapeutic targets**

**Na Xu, Baohong Li, Yong Liu, Cui Yang, Siqi Tang, William CS Cho, Zunnan Huang**

**Supplementary Table 1**: The detailed characteristic differed between low- and high-risk patient samples from GEO database.

|  | **Low risk (n=31)** | **High risk (n=31)** | **Total (n=62)** | **Feature** |
| --- | --- | --- | --- | --- |
| **Survival status** |  |  |  |  |
| Lived | 7 (22.6%) | 16 (51.6%) | 23 (37.1%) | **\** |
| Death | 24 (77.4%) | 15 (48.4%) | 39 (62.9%) |  |
| **Survival time(months)** |  |  |  |  |
| Mean (SD) | 46.4 (28.7) | 30.4 (18.4) | 38.4 (25.2) | **\** |
| Median [MIN, MAX] | 44 [10,120] | 26 [3,74] | 33.5 [3,120] |  |
| **Grade** |  |  |  |  |
| G1/G2 | 11 (35.5%) | 7 (22.6%) | 18 (29.0%) | **\** |
| G3 | 20 (64.5%) | 24 (77.4%) | 44 (71.0%) |  |
| **Lymph node status** |  |  |  |  |
| negative | 24 (77.4%) | 18 (58.1%) | 42 (67.7%) | **\** |
| positive | 7 (22.6%) | 13 (41.9%) | 20 (32.3%) |  |
| **LAMP2** |  |  |  |  |
| Mean (SD) | 10.4 (0.6) | 10.2 (0.5) | 10.3 (0.6) | **▼** |
| Median [MIN, MAX] | 10.3 [8.7,11.5] | 10.2 [8.9,11.1] | 10.2 [8.7,11.5] |  |
| **DPP4** |  |  |  |  |
| Mean (SD) | 6.9 (1.2) | 6.3 (0.8) | 6.6 (1.1) | **▼** |
| Median [MIN, MAX] | 6.7 [4.5,10.3] | 6.3 [4.5,8.3] | 6.5 [4.5,10.3] |  |
| **CAV1** |  |  |  |  |
| Mean (SD) | 10.1 (1) | 9.7 (0.7) | 9.9 (0.9) | **▼** |
| Median [MIN, MAX] | 10.2 [7.4,12] | 9.8 [8.2,10.9] | 10 [7.4,12] |  |
| **SLC7A11** |  |  |  |  |
| Mean (SD) | 6.9 (0.6) | 7 (0.5) | 6.9 (0.5) | **▲** |
| Median [MIN, MAX] | 7 [5.6,8.5] | 6.9 [6.3,8.4] | 6.9 [5.6,8.5] |  |
| **TF** |  |  |  |  |
| Mean (SD) | 6.7 (1.4) | 7.4 (2) | 7 (1.8) | **▲** |
| Median [MIN, MAX] | 6.7 [3.4,10.7] | 6.7 [4.7,12.6] | 6.7 [3.4,12.6] |  |
| **HMGB1** |  |  |  |  |
| Mean (SD) | 12.8 (0.2) | 13 (0.3) | 12.9 (0.3) | **▲** |
| Median [MIN, MAX] | 12.7 [12.4,13.3] | 13 [12.6,13.6] | 12.9 [12.4,13.6] |  |
| **CDKN2A** |  |  |  |  |
| Mean (SD) | 8.5 (1.2) | 9.3 (1.3) | 8.9 (1.3) | **▲** |
| Median [MIN, MAX] | 8.2 [6.8,11.1] | 9.6 [7.1,11.1] | 8.7 [6.8,11.1] |  |
| **ZFP69B** |  |  |  |  |
| Mean (SD) | 5.4 (1) | 5.6 (0.9) | 5.5 (1) | **▲** |
| Median [MIN, MAX] | 5.4 [2.8,7.1] | 5.8 [2.8,7.3] | 5.5 [2.8,7.3] |  |
| **ELAVL1** |  |  |  |  |
| Mean (SD) | 10.5 (0.3) | 10.8 (0.3) | 10.6 (0.3) | **▲** |
| Median [MIN, MAX] | 10.4 [10,11.3] | 10.8 [10.4,11.6] | 10.6 [10,11.6] |  |
| **HELLS** |  |  |  |  |
| Mean (SD) | 4.2 (1.5) | 6.2 (1) | 5.2 (1.6) | **▲** |
| Median [MIN, MAX] | 3.8 [1.8,7.8] | 6.3 [3.9,8.1] | 5.5 [1.8,8.1] |  |
| **PSAT1** |  |  |  |  |
| Mean (SD) | 8.8 (1.1) | 9.2 (1.1) | 9 (1.1) | **▲** |
| Median [MIN, MAX] | 8.6 [6.8,11.3] | 9 [6.7,11.1] | 9 [6.7,11.3] |  |
| **ASNS** |  |  |  |  |
| Mean (SD) | 9.2 (1) | 9.4 (0.9) | 9.3 (0.9) | **▲** |
| Median [MIN, MAX] | 9.2 [7.7,11.1] | 9.4 [7.2,11.3] | 9.3 [7.2,11.3] |  |

Note: **▲** A gene calculated to be upregulated in the high-risk group from GEO database is consistent with that in TCGA database;

**▼** A gene calculated to be downregulated in high-risk group from GEO database is consistent with that in TCGA database;

**▲** A gene calculated to be upregulated in the high-risk group from GEO database which differs from the TCGA database;

**▼** A gene calculated to be downregulated in the high risk-group from GEO database which differs from the TCGA database.

TCGA database: the Cancer Genome Atlas database (https://portal.gdc.cancer.gov/)

GEO database: the Gene Expression Omnibus database (https://www.ncbi.nlm.nih.gov/geo/)

SD: Standard Deviation
